# NIEHS Extramural Global Environmental Health Portfolio: Opportunities for Collaboration

**DOI:** 10.1289/ehp.11323

**Published:** 2008-03-06

**Authors:** Christina H. Drew, Martha I. Barnes, Jerry Phelps, Bennett Van Houten

**Affiliations:** Program Analysis Branch, Division of Extramural Research and Training, National Institute of Environmental Health Sciences, National Institutes of Health, Department of Health and Human Services, Research Triangle Park, North Carolina, USA

**Keywords:** global health, partnerships, science assessment

## Abstract

**Background:**

Global environmental health has emerged as a critical topic for environmental health researchers and practitioners. Estimates of the environmental contribution of total worldwide disease burden range from 25 to 33%.

**Objective:**

We reviewed grants funded by the National Institute of Environmental Health Sciences (NIEHS) during 2005–2007 to evaluate the costs and scientific composition of the global environmental health portfolio, with the ultimate aim of strengthening global environmental health research partnerships.

**Methods/Results:**

We examined NIEHS grant research databases to identify the global environmental health portfolio. In the past 3 fiscal years (2005–2007), the NIEHS funded 57 scientific research projects in 37 countries, at an estimated cost of $30 million. Metals such as arsenic, methylmercury, and lead are the most frequently studied toxic agents, but a wide range of stressors, routes of exposure, and agents are addressed in the portfolio.

**Conclusions:**

The portfolio analysis indicates that there is a firm foundation of research activities upon which additional global environmental health partnerships could be encouraged. Current data structures could be strengthened to support more automated analysis of grantee information.

Global environmental health (GEH) has evolved into a critical topic for environmental health researchers and practitioners. Emergent issues—such as health effects of climate change and electronic waste disposal—have joined more familiar GEH concerns such as urban air pollution; indoor air pollution from cooking with solid fuel; exposure to water contaminated with natural or man-made pollutants such as lead, arsenic, polychlorinated biphenyls, or methylmercury; contaminated land; and traffic injuries. Given that health and disease status result from a complex process of environmental exposure, genetic predispositions, and social circumstances, determining the extent of the environmental contribution of global disease is challenging. Environmental factors play an important etiologic role in at least 5 of the top 10 causes of mortality around the world [World Health Organization (WHO) 2007]. Estimates of the environmental contribution of total worldwide disease burden are 25–33% (Smith et al. 1999). Estimates of environmental burden for specific diseases vary (Table 1) (Smith et al. 1999; WHO 2002).

Many international and U.S.-based organizations are addressing GEH issues. For example, the WHO, the United Nations Environment Programme, the United Nations Children’s Fund (UNICEF) and the United Nations Development Programme (UNDP) have invested in a wide range of programs that conduct research, provide resources, and develop and distribute tools to address and mitigate the environmental contributions to disease (UNDP 2008; UNICEF 2008; WHO 2008). The Millennium Ecosystem Assessment and the Intergovernmental Panel on Climate Change (IPCC) are helping to characterize the expected health implications of global climate change (IPCC 2007; Millennium Ecosystem Assessment 2008). U.S.-based agencies and foundations, such as the U.S. Agency for International Development (USAID), the National Institutes of Health’s (NIH) Fogarty International Center, the Bill & Melinda Gates Foundation, and many more, are also investing resources to examine the distribution and severity of disease, to prevent exposures, and to develop interventions to improve health (Bill & Melinda Gates Foundation 2008; Fogarty International Center 2008; USAID 2008).

A common theme to emerge from these efforts is that collective action is needed. Researchers may be interested in developing partnerships, but information about existing activities is often scarce. The National Institute of Environmental Health Sciences (NIEHS) has a long tradition of supporting research to address GEH concerns. Indeed, GEH is a central component of the NIEHS Strategic Plan (NIEHS 2006). The analysis reported here was conducted as part of an effort to shape the future direction of NIEHS’s GEH program. It is important to catalog what research is funded in order to understand potential gaps, and also to share information that will foster partnerships. We analyzed the portfolio of NIEHS-funded research grants [fiscal years (FYs) 2005–2007] to determine the breadth of topics being addressed by these grants, as well as the institute’s overall investment levels.

## What is Global Environmental Health?

GEH can be defined in many ways. For the purposes of this analysis, we chose a fairly narrow definition in order to identify major research projects in the NIEHS grant portfolio that could form the basis for research partnerships. Thus, we defined GEH as research occurring outside the United States that either included evaluation of a foreign population or collected environmental samples from a foreign location. Our definition of foreign population included any research involving human tissue samples (e.g., urine, blood, DNA), clinical research with individuals (e.g., disease treatment), or community work with various populations (e.g., public health education interventions). The definition did not include collaborations with foreign scientists for which there was no significant foreign population component. Although international conferences and training activities have the potential for exchanging information and building partnerships, we excluded them to maintain focus on scientific research activities.

Our definition of GEH excluded work being performed solely in the United States on issues that can arguably have a global impact. For example, it is possible to make the case that all mercury work could be defined as part of the GEH portfolio because the movement of this compound frequently originates in industrialized societies, travels throughout the world, and causes adverse effects. Although the NIEHS funds a considerable amount of mercury research, projects were excluded from this analysis if research occurred solely within the United States. Harmful algal blooms represent another example of a potential GEH concern that was excluded. Plumes forming in oceanic currents off the shores of the United States are potentially affecting foreign populations in Canada, the Caribbean, and Central America. The NIEHS funds four Centers for Oceans and Human Health, which are all looking at various aspects of the development, distribution, and health effects of harmful algal blooms (NIEHS 2007a). However, we did not include these centers and other research on harmful algal blooms because this research is being conducted largely by U.S.-based researchers.

## Identifying the GEH Portfolio

Once we established a definition of GEH, we set about identifying grants that met that definition. Unfortunately, current NIH data structures lack an automated way to query for information needed to meet the definition, and other investment databases, such as the RAND Corporation’s RadiUS database (RAND Corporation 2007), do not include research and development information for areas outside in the United States. The Foreign Tracking System (FTS), a database of NIH’s foreign investment housed at the Fogarty International Center (2007) and the Information for Management, Planning, Analysis, and Coordination (IMPAC) II data system were the primary data sources for the project (NIH 2007). A complex and time-consuming search strategy was required to identify the appropriate grants.

The IMPAC II data system provides grant tracking and management data for extramural research grants funded by the NIH. (The NIH conducts research directly, and also funds other organizations to conduct research; these types of research are termed intramural and extramural, respectively.) The main data elements used in the analysis were the abstracts submitted with the funded applications and the most recently updated budget tables, both accessible through IMPAC II (NIH 2007). We used the FTS primarily for subproject budget information. Occasionally, specific aims from application files were consulted for additional details. In general, the unit of analysis presented here is a single grant. However, about 20% of the portfolio is composed of projects that are components of larger multiproject grants (such as Superfund multiprogram projects or Environmental Health Science Core Centers). In such cases, we treated subprojects as a single project (most address similar environmental contaminants), but we developed budget estimates using subproject data to provide a more accurate estimate of investment.

Initially, we identified a collection of potential GEH grants by selecting any of the following criteria: *a*) any grant encoded in IMPAC II as a “foreign grant” with budget dollars paid in FY 2005, 2006, or 2007; *b*) any grant identified by the FTS; or *c*) any grant identified by NIEHS program officers as having a foreign cohort or sample collection. The initial list of 135 candidates was further narrowed based on the definition criteria above. In other words, collaborations providing support only for international colleagues (but no samples or cohorts) were excluded, as were foreign conferences, contracts, and work done at a U.S. university on foreign data sets. The final list was reviewed by NIEHS program officers in the Division of Extramural Research and Training.

**Figure 1:**
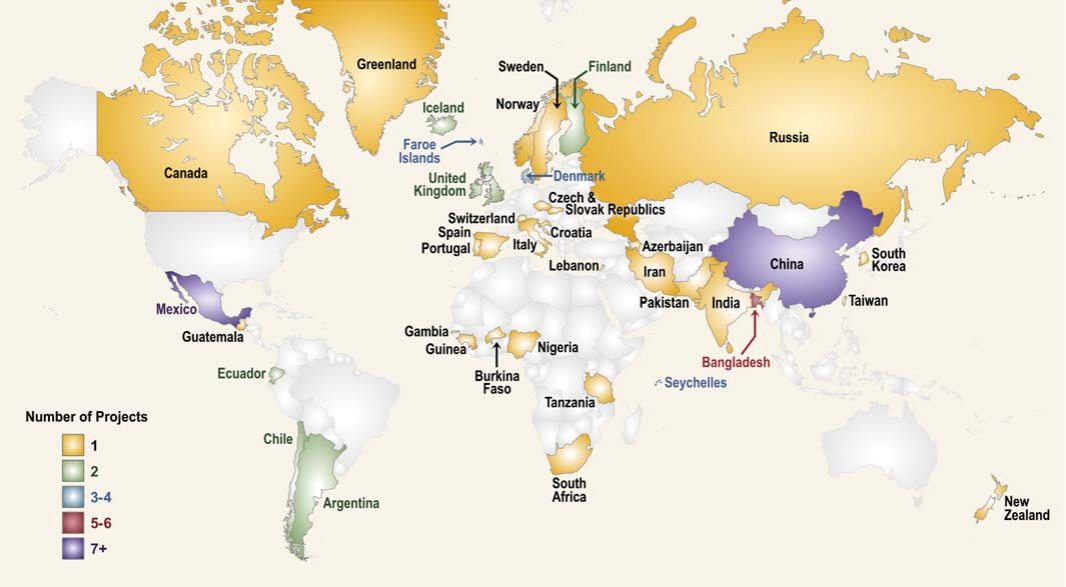
The NIEHS extramural GEH portfolio during FYs 2005–2007. The portfolio includes 57 projects in 37 countries across the globe. Greenland and Faroe Islands are shown as separate entities, but they are officially Danish territories and thus are not counted as separate countries.

Ultimately, we identified 57 extramural projects in 37 countries outside the United States as having a GEH component during FYs 2005–2007 (Figure 1; see also Supplemental Material, Table S1, available online at http://www.ehponline.org/members/2008/11323/suppl.pdf). The approximate cost of these grants (or their relevant components) is $30 million over the 3-year period, with annual breakdowns of about $6.3 million in FY 2005, $12 million in FY 2006, and $11.7 million in FY 2007. For comparison, the entire extramural NIEHS portfolio of projects funded in FY 2007 was approximately 890 projects at a cost of $380 million (including Superfund programs), and the GEH portfolio comprised about 3% of that total cost. Most of the GEH grants are individual investigator-initiated (R01) projects. Approximately 72% of the funds expended in the GEH program were delivered through the R01 mechanism.

## Scientific Fields Represented in the GEH Portfolio

**Figure 2:**
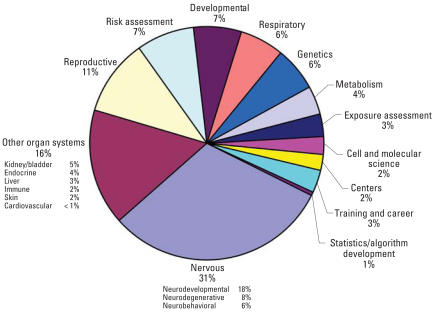
NIEHS GEH portfolio, with the primary science codes represented in the 2005–2007 portfolio shown as a percentage of total dollars. The primary science codes have been grouped into categories, and percentages were calculated based on the combined total cost of the grants (or subprojects) in each of the major categories. The total combined cost for the period is estimated at just over $30 million.

Next we reviewed the science contained in the grants, relying on two analytical approaches. First, we assessed the NIEHS science codes within the GEH portfolio. The current set of science codes was developed starting in 2005, and are specific to NIEHS [additional information about the science codes is available online (NIEHS 2007b)]. The codes range from 01 to 99, although not all numbers are currently assigned. Each grant has a single primary science code embedded in its IMPAC II record. Currently, science codes are primarily assigned by organ systems, disease outcomes, or mechanism (e.g., DNA repair) rather than by exposure agent (Supplemental Material, Table S2, available online at http://www.ehponline.org/members/2008/11323/suppl.pdf). When multiple systems are studied in a single grant, the grant is given the primary code that most closely matches the majority of the research. A secondary science code may also be given, but is not routinely assigned. Based on the assignment of primary science code, areas of science receiving the most funds in the GEH portfolio are the nervous system (31%), the reproductive system (11%), developmental issues (7%), and the respiratory system (6%) (Figure 2).

**Figure 3:**
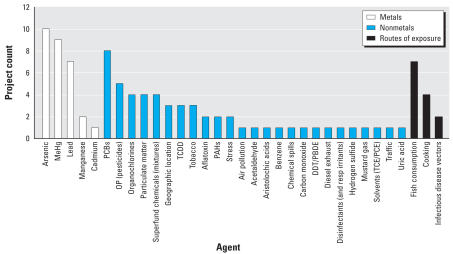
Exposure agents in the NIEHS GEH portfolio (2005–2007). Abbreviations: DDT, dichlorodiphenyl-trichloroethane; MeHg, methylmercury; OP, organophosphate; PAHs, polycyclic aromatic hydrocarbons; PBDE, polybrominated diphenyl ether; PCBs, polychlorinated biphenyls; PCE, perchloroethylene; resp, respiratory; TCDD, tetrachlorodibenzodioxin; TCE, trichloroethylene. We hand coded the grant abstracts to identify exposure agents listed. If a grant focused on more than one exposure agent, each instance was counted multiple times. Thus the sum total of the exposure agents named (94) is greater than the total number of projects in the portfolio (57). As the most common toxicants studied, metals are distinguished from nonmetals and exposure routes.

Additionally, we analyzed the breadth of exposure agents and routes of exposure represented in the portfolio. Because this information is not coded systematically in the IMPAC II data system, the analysis was performed by hand, based on the investigator-written abstracts and titles for the grants; we also used subproject abstracts as a data source for multiprogram project grants. Figure 3 illustrates the wide range of exposure parameters represented in the portfolio. Metals are the most studied exposure agent, with arsenic, methylmercury, and lead being the most common metals studied. Among nonmetal agents, polychlorinated biphenyls, organophosphate and organochlorine pesticides, particulate matter, and mixtures were the next most frequently studied. Several studies specifically focused on consumption of fish or inhalation of smoke from cooking fires as potential routes of exposure.

## GEH Portfolio Analysis Challenges

Determining the total overseas investment in GEH is difficult. The data in IMPAC II budget tables do not track actual overseas investment in a way that is easily extractable, so the total project dollars associated with a grant or relevant subproject(s) are reported. However, it is likely incorrect to assume that this total sum is actually being spent overseas. The program costs reported here are therefore likely to be an overestimate of GEH portfolio investment during 2005–2007. Nevertheless, important components of these grants and projects occur within the U.S. border, and it would be somewhat arbitrary to only count funds spent overseas.

Another limitation was use of the primary science code designation as the main way to assess the science contained within a grant or subproject. On the one hand, having each grant (or subproject) assigned to a single code is a helpful way to understand the main emphasis of a particular grant and simplifies the reporting process. On the other hand, a rigid classification of activities into a single category oversimplifies the complexity of most grants. We attempted to address this challenge by hand coding the exposure agents, allowing as many categories for each grant as needed. However, the time commitment required to hand code limited our ability to evaluate and code other factors of interest, such as life stage, sex, genetic aspects, or sample type. Moreover, determining the costs associated with each exposure agent presents another challenge: It would be almost impossible to assign portions of dollars expended within a grant on the diverse range of potential toxic exposures with any degree of consistency.

All efforts were made to apply a systematic coding approach for the environmental contaminants and routes of exposure. However, codes were developed inductively based on the data encountered, and not matched to any consistent structured vocabulary [such as the Medical Sub-Headings (MeSH) vocabulary developed by the National Library of Medicine]. The NIEHS differs from other NIH organizations in the breadth of topics that are emphasized in its research portfolio. Research can be categorized by toxin, mechanism of action, organ system or disease, or route of exposure. Many research projects address several of these categories but rarely address all of them. Different audiences are interested in different categories. The system of assigning at most two science codes can oversimplify the focus of a project. However, the IMPAC II database does not easily support the designation of a grant into multiple portfolios. This limits our ability to quickly obtain information about the breadth and depth of our activities in all areas. The challenge is particularly acute for GEH because these grants are not easily identifiable with simple key words. Given that all grants to the NIH are now submitted electronically, automated systems for assigning and tracking portfolios are now conceivable and are being pursued actively at the NIEHS.

Additionally, external factors sometimes cause the scope and content of a grant to change over time. Changes are tracked and cataloged in the annual progress reports, but the original abstract may be as much as 5 years old, and thus may be out of date. For example, one study that originally proposed to conduct research in Peru and Japan is now studying populations in Peru and China. We relied on program officers’ knowledge of their grantees’ activities to help us identify instances where the abstract was not up to date.

Finally, restricting the analysis to titles and abstracts, the text-searchable grant components in IMPAC II, may have limited the analysis in a number of ways. First, additional exposure routes or agents could have been described in the methods or specific-aims sections of individual applications. Second, important research study parameters were also omitted because they are not typically included in abstracts. Examples of parameters that could provide useful details for developing partnerships include cohort population parameters (size, ethnicity, sex, animal model strain); the lifestage of exposure/disease (prenatal, childhood, adult, elderly); biosamples/tissues collected; exposure media (air, water, food); genes studied; or remediation technology (if any) used to mitigate contamination. Without the ability to search the full text of grant proposals and annual progress reports, analyzing these parameters would require reading individual grants and coding items of interest by hand, or surveying the grantees directly. Both approaches would be laborious and the latter would likely require approval from the U.S. Office of Management and Budget. Again, the recent expansion of electronic grant data could be tapped to provide more a more robust basis for analysis.

## Conclusions

We are all part of the same global environment; contaminants do not recognize national borders. Environmental factors clearly play an important etiologic role in many of the leading causes of morbidity and mortality. Accordingly, GEH research is a growing segment of the overall NIEHS portfolio. A key first step to strengthening the portfolio is to understand what is currently being funded. Existing data tracking systems need to be strengthened to support automated analysis of grantee information. Maximizing the GEH effort also requires that NIEHS leverage its resources with other organizations throughout the world. Many existing research projects represent opportunities for collaboration and partnership. Studies in existing cohorts or biosamples could be expanded to include additional exposure agents or genetic factors. Additionally, new techniques for basic science in other parts of the NIEHS portfolio could be applied to activities occurring in other countries.

In an effort to broaden GEH partnerships, the NIEHS has recently sponsored several workshops. In January 2007, the NIEHS gathered an international panel of scientists in San Francisco, California, to participate in a Global Environmental Health Conference (NIEHS 2007c). This working meeting was designed to provide guidance to the NIEHS about potential research opportunities and strategies as GEH science receives greater emphasis. Participants proposed and ranked research project topics and short-term strategies for conducting research. At another meeting in Mexico City (September 2007), the NIEHS met with 12 international air pollution research experts to discuss the breadth and depth of different air pollution studies around the world, and to assess the feasibility of comparing and pooling data to better understand the diverse clinical responses and genetic susceptibility to air pollution exposure across different populations (NIEHS, unpublished data). Additional efforts, such as a forthcoming NIEHS website focused on GEH, will continue to provide new avenues to share the results of our research and build partnerships.

## Figures and Tables

**Table 1:** WHO statistics on the environmental burden of disease.

Environmental hazard	Estimated disease burden
Unsafe water sanitation and hygiene	3.1% of deaths (1.7 million) worldwide
Ambient air pollution	0.8 million (1.4%) deaths, with the burden predominantly in developing countries
Indoor smoke from solid fuels^a^	35.7% of lower respiratory infections 22.0% of chronic obstructive pulmonary disease 1.5% of trachea, bronchus, and lung cancer Also associated with tuberculosis, cataracts, and asthma
Elevated blood lead levels in industrialized countries	5% of children still have elevated blood lead levels, with higher rates in children of poorer households
Elevated blood lead levels worldwide	40% of children have blood lead levels > 5 μg/dL 97% of affected children live in developing regions
Climate change	2.4% of worldwide diarrhea 6% of malaria in some middle-income countries 7% of dengue fever in some industrialized countries

Data from WHO (2002).

a Nearly half the world cooks with solid fuels.
